# A thank you to our 2024 reviewers and update on our inclusion and diversity commitments

**DOI:** 10.1016/j.lana.2025.101025

**Published:** 2025-02-14

We extend our heartfelt gratitude to each one of you who contributed as a reviewer to *The Lancet Regional Health—Americas* in 2024 for your invaluable contributions and expertise in the peer review process. Your diligent efforts and insightful feedback are the cornerstone of our journal's success, ensuring the publication of high-quality, impactful research. We recognize that our achievements are intimately linked to your unwavering support and commitment.

Aligned with our mission to give voice to the Americas region, we strive for a peer review process that includes voices and perspectives from the region, who we believe are best placed to provide contextualized feedback. In 2024, 53.5% of our reviewers were from the Americas region, 58.6% from North America and 41.4% from Latin America and the Caribbean (LAC). Regionally placed reviewers are crucial for providing insights rooted in the local context, which enriches the relevance and impact of the research we publish. We recognize the need to continue to improve our reviewer representation from the region, especially from LAC countries.

As we strive for excellence, we are also committed to promoting diversity and inclusivity within our reviewer community. In 2024, from the 817 reviewers who accepted an invitation to review, 44.7% self-identified as a woman, 50.6% as a man, 0.5% as non-binary or gender diverse, and 4.3% preferred not to disclose. While these figures suggest we are on the right path, we remain dedicated to continuing our efforts towards improvement.

A significant initiative across all Lancet journals has been the update to our Editorial Manager submission portal with Equity, Diversity, and Inclusion (EDI) personal classifications, launched in September 2024. Users can now voluntarily assign these EDI classifications to submitted manuscripts or to their individual accounts to indicate their expertise. We anticipate that this feature will enhance reviewer searches and contribute to our broader diversity and inclusion goals. For more information on how to update the personal classifications in your Editorial Manager account, please contact *The Lancet Regional Health–Americas* at americas@lancet.com.

We firmly believe that peer review is a key part of gatekeeping scientific integrity and a task that deserves much greater recognition. The dedication you bring to your reviews not only upholds the scientific integrity of our journal but also fosters the growth and development of the broader scientific community. Thank you once again to all the 817 reviewers from 63 different countries for your exceptional contributions and dedication to our journal. Together, we can continue our mission to publish the best science to advance health in the Americas region and globally.

Fernando Abad-Franch

Gabriel Abarca-Brown

Marie A Abate

Romesh Abeysuriya

Naeemah Abrahams

Dmitry Abramov

Gerardo Acosta-Jamett

Victor Adekanmbi

Aniruddha Adiga

Kwabena Agbedinu

Sergio Agudelo-Pérez

Agnes C Agunos

Bilal Ahmed

Samir Al-Adawi

Margarita Alegria

Gregory L Alexander

Márlon Aliberti

Lao-Tzu Allan-Blitz

Bernardo Almeida

Walter Ricardo Almirón

Flávia Jôse Oliveira Alves

Shanthi Ameratunga

Sohrab Amiri

Alexa Anderson

Benjamin Anderson

Melissa Andrew

Josep Antó

Neftali Eduardo Antonio-Villa

Joshua Anzinger

Makoto Aoki

Catalina Arango-Ferreira

Edward Araujo Júnior

Elizabeth Arias

Jean-Benoît Arlet

Tarik Asselah

Paul Auwaerter

Aziz Ullah Awan

Ricardo Ayala

Beatriz Paulina Ayala Quintanilla

Sebastian Rolando Ayala-Beas

Priti Azad

Raimunda Azevedo

Luis Bahamondes

Manisha Bahl

Shasha Bai

Miklosh Bala

Edward P Balaban

Paola Ballotari

Robert Bals

Connor Bamford

Maciej Banach

Ananda Bandyopadhyay

Jessica A Barber

Magali Barbieri

Jennifer Lynn Barkin

Joaquim Barreto

Imke Heleen Bartelink

Alfred Bartolucci

Francisco Bastos

Mayara Lisboa Bastos

Philip Batterham

Nancy Baxter

Robert A Bednarczyk

Jasper Been

Omar Yaxmehen Bello-Chavolla

Julie Benbenishty

James Bentham

Irina Bergenfeld

Cari Berget

Antonio Bernabe-Ortiz

Filipe Andrade Bernardi

Thaís Zamboni Berra

Cheryl Lynn Beseler

Conceição Bettencourt

Nathan Beucler

Zulfiqar Bhutta

Sheila Bird

Madelyne Bisby

Kirsten Black

Cari Bogulski

Archith Boloor

Wyllians Borelli

Jacob T Borodovsky

Rohan Borschmann

Ariane Boumendil

Erick Boy

Jeremy Braithwaite

Lars Brännström

Liz Brewster

Elizabeth Brickley

Matthijs Brouwer

Joe Brown

Amanda S Bruegl

Silvio Brusaferro

Christoph Buck

Nassib Bueno

Romina Buffarini

Danilo Buonsenso

Analía Burgueño

Joseph Burzynski

Siddappa Byrareddy

Baltica Cabieses

Xiaoling Cai

Vera Caine

Clara Calvert

Cristina Calvo

Miguel Camafort-Babkowski

M Constanza Camargo

Jackie Campbell

Jeffrey I Campbell

Alejandra J Cantoral

Martin Caraher

Rodrigo Carmo

Adam Carrico

Jennifer Carroll

María Carroza-Escobar

Ben Carter

Abigail Casas-Muñoz

Yvan Caspar

Tatiana Castro Abreu Pinto

Thiago Cerqueira-Silva

Juraci Cesar

Savaş Ceylan

Philip Chan

Ram Hari Chapagain

Mariana Chavez-Villa

Han-Yang Chen

Jun Chen

Wei Chen

Yingyao Chen

Ramsey Cheung

Pedro Paulo Chieffi

Shu-Ling Chong

Esther Choo

Christos Chouaid

Kurt D Christensen

Katerina Christopoulos

Mary Catherine Clarke

Ralf Clemens

Lindsay J Collin

Jean-Frédéric Colombel

Stephen Congly

Anna Conway

Andrew Copas

Rosie Cornish

Diogo Corrêa

Andrea Cortegiani

Jorge Cortes

Maria Laura Costa

Silvia Costa

Benjamin Cowling

Gerard Criner

Aurelio Cruz-Valdez

Amanda Cunha

Maria Curado

Thomas Czypionka

Jesse Lopes da Silva

Thiago da Silva

Chia-Yen Dai

Paulo Tarso Dalcin

Florence Dallo

Rakhi Dandona

M Carolina Danovaro-Holliday

Mike Daube

Stéphane Dauger

Miranda Davies-Tuck

Claudia Dávila

Kelvin de Andrade

Marianna De Camargo Cancela

Patrícia F de Fragas Hinnig

Marcelo Teixeira de Holanda

Marlieke de Kraker

Xavier de Lamballerie

Adam de Smith

Esther de Vries

Kora DeBeck

Edward Dee

Felipe Delpino

Shihan Deng

David Denning

Salil Deo

Claudia Der-Martirosian

Delan Devakumar

Daniel Diaz

Javier Díaz

Luis Antonio Díaz

Fredi Diaz-Quijano

Joseph Dieleman

John Dillon

Tao Ding

Rhoel Dinglasan

Matthew Dodd

Laura Dodge

Safi Dokmak

Rosa Maria Domingues

Bryan Donald

Jianzeng Dong

Caetano C Dorea

Ryan S Doster

Kirk Douglas

Nicholas Douglas

Ana Paula Drummond-Lage

Sérgio Jose Dubner

Roy William Roland Dudley

Lisa M Dunkle

David Durand Vara

Laura Dwyer-Lindgren

Raoudha Dziri

Andrew David Eaton

Andrey Egorov

Anna Mia Ekstrom

Moawia Elhassan

Katherine Ellingson

Nancy Margaret Endersby-Harshman

Babak Eshrati

Ryan Essex

Elizabet Estallo

Guadalupe Estrada-Gutierrez

D Gareth Evans

Elizabeth A Evans

Jessica Fairley

Giuseppe Fallara

Hui Fan

Eric Faragher

Tara J Faraoni

Forough Farrokhyar

Lydia A Fein

Ariadna Feliu Josa

Zhichun Feng

Michelle Fernandez

Pedro Fernández-Soto

Paula A Ferrada

Aline Ferreira

Angela Cristine Bersch Ferreira

Marica Ferri

Ceu Figueiredo

Teresa Filipponi

Walter Flores

Nathan P Ford

Diego A Forero

Sandrine Fosse-Edorh

Marcondes França Jr

Anna Freni Sterrantino

Francesca Frentiu

Flavio Fuchs

Ary Gadelha

John N Galgiani

Miguel Galícia

Barbara C Galland

Sandra Gallego

James Galloway

Tingting Gao

Guillermo Garcia-Garcia

Scott Lyell Gardner

Pankaj Garg

Ximena Garzón-Villalba

Rebecca Geary

Ayla Gerk

Abdolmajid Ghasemian

Nicolae Gică

Wendy Gifford

Sheena Gilbert

Thomas E Gill

Marta Giovanetti

Robert Gish

Sherry Glied

Alyssa Golden

Shira Goldenberg

Delia Goletti

Fabio Gomes

Kelly Lucy Guimarães Gomes

Flávia Gomes-Sponholz

Scarlett Lin Gomez

Jorge Enrique Gomez-Marin

Bruno Gonçalves

John Mario Gonzalez

Jessica González Gutiérrez

Alejandro Gonzalez-Aquines

Maria Carlota Gonzalez-Bedat

Catalina González-Uribe

Laura B Goodman

David Goodman-Meza

Alessandra Goulart

Christopher Green

Chris Gregory

John Gregson

Lionel Gresh

Jonathan Grigg

Anneke Grobler

Erik Groessl

Alessandra Agnese Grossi

Sebastian Gruhn

Humberto Guanche Garcell

Alessandra C Guedes

Jorge Augusto De Oliveira Guerra

João Guerreiro

Thais Guimaraes

Rafael Lima Guimarães

Raphael Guimarães

Xu-Guang Guo

Tahar Hajri

Wayne Hall

David Halpin

Ali Hamedani

Ariel Hammerman

Jin Han

Shuping Han

Yueh-Ying Han

Ivan Hanigan

Jessica Harding

Michael Harhay

Diane Harper

Zachary Hass

Joanne Haviland

Benjamin T Hayes

Yulei He

Oskari Heikinheimo

Andrés Henao-Martínez

Claire Henderson

Jaymie Ang Henry

Rolando Herrero

Fátima Higuera-De-La-Tijera

Michael Hill

Daniel J Hoffman

Paul Holloway

Christy L Hom

Lisa Horowitz

Charles Horsburgh Jr

Christopher Horvat

M Mahbub Hossain

John Howard

Emily Howerton

Yu-Hsiang Hsieh

Qing-Hai Hu

Chuanchin Huang

Chuiguo Huang

Lin Huff-Corzine

Silvie Huijben

Keith Humphreys

Sally Hunsberger

Jamil Hussain

Francisco Ibanez-Carrasco

Pedro Iglesias

Usman Nurudeen A Ikumapayi

Hiroyuki Inose

Ana-Maria Iosif

Stephanie Irving

Abdurrahman I Islim

Gaetano Isola

Kentaro Ito

Katherine M Iverson

Anand Iyer

Alane Izu

Thiago Jacomasso

María Jaimes-Reyes

Dorota M Jamrozy

José Januario

Johan Jendle

Degu Jerene

Fanpu Ji

Xiaofan Jia

Emily E Johnston

Jeb Jones

Vita Jongen

K S Joseph

Kathie Ann P Joseph

Boyoung Joung

Steven Julious

Jihoon Jung

Monica Jung

Robert Justo

Anna Kalbarczyk

Kevin F Kamis

Vinicius Kannen

Theodoros Karantanos

Sayyeda Karim

N Yu Kashirskaya

Ilan Katz

Amirreza Kazemikhasragh

Christine Kelly

Mark Kelson

Zoe Kelson

Carl Kendall

Ali Khashan

Utsha Khatri

Ardeshir Khosravi

Rikke Munk Killingmo

Carina King

Patrick Kiser

Jules K Koffi

Susanna Kola-Palmer

Evangelos Kontopantelis

Tomomi Kotani

Bernardo Javier Krause

Alexander Kreuter

Evangelos Kritsotakis

Candyce Kroenke

Daniel J Kruger

Kiersten Kugeler

Mohan Kumar

Carlo La Vecchia

Alessio Lachi

Simone Ladeia-Andrade

Francisco Lai

Adeyinka O Laiyemo

Theresa Lamagni

Sven Arke Lang

Alison Laufer Halpin

Carl Lavie

Julia Lechuga

Gwenyth Lee

Jiun Lee

Wei Lin Lee

Herman Lelieveldt

Maddalena Lettino

Wai Leung

John Leventhal

Anna Levin

Yafit Levin

Stephen Levine

Anders Lewén

Dan Li

Jinghua Li

Jingjing Li

Jingxin Li

Suzanne C Li

Bingyu Liang

Donna Lin

Leesa Lin

Jue Liu

Ruiling Liu

Yiran Liu

Gill Livingston

Marcela Lizano

Andrea Llera

Ellen Løkkegaard

Xiao Long

Miriam Longo

María Soledad López

Raúl López-Antón

Alejandra López-Gómez

Milena Lopreite

Camila Lorenz

Angela Loyse

Ivan Lozada-Martinez

Chan Lu

Giancarlo Lucchetti

Ramon Luengo-Fernandez

Isaac Luginaah

Hao Luo

Suzanne MacFarland

Anne E MacFarlane

Priscila Machado

Peter Macharia

Rafael Maciel-de-Freitas

James Macinko

Emily Lai Ho MacLean

Peter MacPherson

Lipi B Mahanta

Phuong Mai

Heimo Mairbäurl

Lung-Yi Mak

Linda Malan

Monica Malta

Corinne Mandin

Mohammad Ali Mansournia

Chris R Marcellino

Michele Marchioni

Ulrich Marcus

Têmis Maria Félix

Caitlin Martin

Javier Martín

Marianne Martinello

Chenia Caldeira Martinez

Pablo Martinez-Camblor

Paulo Ricardo Martins-Filho

Luis Paulo Gomes Mascarenhas

Joanna Maselko

Paulo Eduardo Luiz Mattos

Margaret McConnell

Donald McIntire

Danette Waller McKinley

Anna McNaughton

Mauro Mediano

Reinier Meester

Pallavi Mehta

Yatin Mehta

Fiona Mensah

Anwar Merchant

Stefan Meyer

Martina Micai

Joseph R Mihaljevic

Nkuchia M'ikanatha

Mark Miller

Rachel Miller

James Milner

Guadalupe Miranda-Novales

Adrienne Mishkin

Seyed Moghadas

Viswanathan Mohan

Jean-Pierre Molès

Sabrina Molinaro

Anna Molto

Martha Montgomery

Fabio Moraes

Gláucia Maria Moraes de Oliveira

Javier Morán-Martínez

Carla Moreira

Thereza Maria Magalhães Moreira

Jonna L Morris

Sagori Mukhopadhyay

Amy Mulick

Pricila Mullachery

Kerri Anne Mullen

Malaisamy Muniyandi

Gwen A Murphy

Joseph Murray

Felipe Murta

Vincent Mysliwiec

Farooq Naeem

Mohsen Naghavi

Gustavo J Nagy

Harish Nair

Kumar Narayanan

Sarah H Nash

Sharifa Nasreen

Antonio Paulo Nassar Junior

Adriana Navarro-Sainz

Mytien Nguyen

Mya Myat Ngwe Tun

Claire Nightingale

Zlatko Nikoloski

Sebastián Fernando Niño-Ramírez

John Norrie

Bernard North

Jean Jacques Noubiap

Letícia Nunes Campos

Thomas Nyström

Atsushi Oba

Katie O'Brien

Vanessa M Oddo

Emmanuel Okoye

Gustavo Olaíz-Fernández

Vincenzo Oliva

Andre Oliveira

Claúdia Oliveira

Gabrielle Oliveira

Gustavo Bernardes De Figueiredo Oliveira

Isadora Sousa de Oliveira

Joseli Oliveira-Ferreira

Carlos Alberto Ordóñez

Eduardo Ortiz

Edgar Ortiz-Brizuela

Jovita Ortiz-Contreras

Bayla Ostrach

Rita Francesca Padoan

Yasna Palmeiro-Silva

Basu Dev Pandey

Nivedha Panneer

Jean-Jacques Parienti

Miguel Parra-Saavedra

Luke Parry

Patrizio Pasqualetti

Angel José Paternina-Caicedo

Caitlin C Patler

Robert Paulino-Ramírez

Márcio Pavan

Martina Pavlicová

Lillian Pazvakawambwa

Marcella Pazzinatto

Sallie-Anne Pearson

Emily Jane Peckham

Nasheeta Peer

Ruben Pérez

Eliseo Pérez-Stable

Juan Pericas

Eduardo Perna

Nandita Perumal

Olumuyiwa James Peter

Salvatore Petta

Praphan Phanuphak

Viani Picchetti

Reinaldo Pierre

Sophie Pilleron

Amber Pirzada

Milan Piya

Laura C Plantinga

Manuel Podestà

Robert Podolsky

André Pontes-Silva

Philip Poortmans

Michael Poulson

Sergio I Prada

Namrata Prasad

Robin Prescott

German Prieto-Rodriguez

Jennifer M Primack

Rui Providência

Nancy Puttkammer

Wenbin Qian

Carlos Quispe-Vicuña

Daniele Raggi

Mathilde Rainard

Usha Ram

Habib Omari Ramadhani

Rebeca Ramis

Jose-Manuel Ramos-Rincon

Angela Rankine-Mullings

Suman Padubidri Nanyam Rao

Maryam Rassouli

Leah Ratner

Shalini Bhargava Ray

Kenneth E Remy

Bernhard Resch

Chandra A Reynolds

Antonio Ribeiro

Arnoldo Riquelme

Chris Rissel

Rocío Rivero

Javier Roberti

Ermane Robin

Thiago Augusto Hernandes Rocha

Marie Rochette

David Rodriguez-Arias

Aarón Rodríguez-Caballero

Ashley Roen

Leonardo Roever

Aleksandra Rogowska

Claudio A Rojas

Slawa Rokicki

Sonia Roman

Anne Iris Romens

Daniel Romer

Regis Rosa

William Rosenberg

David Ross

Franziska J Rosser

Emeline Rougeaux

Nadia Roumeliotis

Abraham Rami Rudnick

Isaac Ruiz

Rossana Ruiz

Piero Ruscitti

Christiana M Russ

Graciela Russomando

Joselyn Rwebembera

Theresa Ryckman

Altaf Saadi

Caroline Sabin

Marc Saez

Luis Sagaon-Teyssier

Mohamed Said

Paulo H N Saldiva

Laura Samuel

Alvaro Sanabria

Lucila Sanchez-Macedo

Héctor Javier Sánchez-Pérez

Nathan Edward Sanders

Clemax Sant Anna

Silvana Santi

Raul Santos

Victor Santos

Gerardo Santos-López

Alan Roger Santos-Silva

Suélen Saraiva

Fred Sarfo

Elizabeth K Schiffman

Alejandro Schijman

Julia P Schleimer

Lavinia Schuler-Faccini

Ana Carolina Sepúlveda-Vildósola

Rowland Seymour

Sana Shahram

Fiona Shand

Rashmi Sharma

Narek Shaverdian

Connor Mc Devitt Sheehan

Affan Shoukat

Sarah A Shulist

Marcos Cláudio Signorelli

Everton Silva

Gisele Silva

Marinella Silva Laport

Mariangela Freitas Silveira

Abigail Simms

Siddharth Singh

Daisy Singla

Rachel Slayton

Audrey Smargiassi

David R Snydman

Paula Soares

Sajid Soofi

Alonso Soto

Hugo Soudeyns

Carlos Souza

Dyego L B Souza

Kerstin Spanhel

Aaron C Spaulding

Bryan Spencer

Katie Spoon

Steven John Stack

Claire Standley

John Staples

Michael Stein

Charles A Stiller

Carl Streed Jr

Garth Strohbehn

Claudio Struchiner

Zhaohui Su

Maneesh Sud

Teiichi Sugiura

Takehiro Sugiyama

Mina Suh

Ying Sun

Karin Sundström

Bernard Surial

Nuntra Suwantarat

John Szumowski

Caroline Tait

Cheuk Chi Tam

Tomoko Tanaka

Weiming Tang

Xiaowei Tang

Zhaohui Tang

Guoyu Tao

Danielle Tatum

Yacob G Tedla

Sylvia Lopes Maia Teixeira

Masanori Terashima

Alexander Testa

Tanayott Thaweethai

Sathish Thirunavukkarasu

Anne Marie Thow

James Thrasher

Luiz Claudio Thuler

Holly Thurston

Steven Tisseverasinghe

Alessandro Tomelleri

Gabriele Tonni

Julio Torales

Kayla N Tormohlen

Jonel Trebicka

Nico Trocmé

Thomas Clement Truelsen

Tim Tsang

Noel Tsui

Lilian Rumi Tsuruta

Cristina Tudoran

Hugo Turner

Rodman Turpin

Leslie Tutty

Brooks V Udelsman

Kohjiro Ueki

Eduardo Undurraga

Ravi Upadhyay

Miguel Urina-Triana

Marcelo Urquia

Charlotte Usselman

Pablo Valente

Patricio Valenzuela

Patricia Valery

Bert Jan H van den Born

Maarten van Wijhe

Mark Varvares

Elsa María Vásquez-Trespalacios

Walter Ricardo Ventura Laveriano

Ximena P Vergara

Elvina Viennet

Anita Villani

Rodolfo Villena

Marcos Da Cunha Lopes Virmond

Sharon Walmsley

Judd Walson

Jann-Yuan Wang

Jianing Wang

Jianming Wang

Nancy Ewen Wang

Yi Wang

Yuanyuan Wang

Zai Wang

Marcia Weaver

Emily Webb

Fernando C Wehrmeister

Guenter Weiss

Paul Weiss

Xuerong Wen

Astrid Louise Wester

Jeffrey K Wickliffe

Arnold Wilkins

Cathleen Elizabeth Willging

Ariel A Williamson

Julie K Wisch

Lawrence Wissow

Ngai Sze Wong

Robert Wong

Caradee Yael Wright

Chi Shin Wu

Juan Xicohtencatl-Cortes

Stanley Xu

Zhiwei Xu

Zhiyuan Xu

Alice Fang Yan

Cheng-Fang Yen

Haibo Yu

Zhenwei Yu

Laura Zambrano

Fengqing Zhang

Jingning Zhang

Panpan Zhang

Ping Zhang

Zefeng Zhang

Jie V Zhao

Rong Zheng

Feng-Cai Zhu

Gianvincenzo Zuccotti

Alice Anne Zwerling

